# Population Genetics of Franciscana Dolphins (*Pontoporia blainvillei*): Introducing a New Population from the Southern Edge of Their Distribution

**DOI:** 10.1371/journal.pone.0132854

**Published:** 2015-07-29

**Authors:** María Constanza Gariboldi, Juan Ignacio Túnez, Cristina Beatriz Dejean, Mauricio Failla, Alfredo Daniel Vitullo, María Fernanda Negri, Humberto Luis Cappozzo

**Affiliations:** 1 Centro de Estudios Biomédicos, Biotecnológicos, Ambientales y Diagnóstico (CEBBAD), Universidad Maimónides, Ciudad Autónoma de Buenos Aires, Argentina; 2 Consejo Nacional de Investigaciones Científicas y Técnicas (CONICET), Buenos Aires, Argentina; 3 Grupo de Estudios en Ecología de Mamíferos, Departamento de Ciencias Básicas, Universidad Nacional de Luján, Luján, Argentina; 4 Sección Antropología Biológica, Instituto de Ciencias Antropológicas (ICA), Facultad de Filosofía y Letras, Universidad de Buenos Aires (UBA), Ciudad Autónoma de Buenos Aires, Argentina; 5 Fundación Azara, Universidad Maimónides, Ciudad Autónoma de Buenos Aires, Argentina; 6 Fundación Cethus, Buenos Aires, Argentina; 7 Laboratorio de Ecología, Comportamiento y Mamíferos Marinos, Museo Argentino de Ciencias Naturales "Bernardino Rivadavia", Ciudad Autónoma de Buenos Aires, Argentina; Fordham University, UNITED STATES

## Abstract

Due to anthropogenic factors, the franciscana dolphin, *Pontoporia blainvillei*, is the most threatened small cetacean on the Atlantic coast of South America. Four Franciscana Management Areas have been proposed: Espiritu Santo to Rio de Janeiro (FMA I), São Paulo to Santa Catarina (FMA II), Rio Grande do Sul to Uruguay (FMA III), and Argentina (FMA IV). Further genetic studies distinguished additional populations within these FMAs. We analyzed the population structure, phylogeography, and demographic history in the southernmost portion of the species range. From the analysis of mitochondrial DNA control region sequences, 5 novel haplotypes were found, totalizing 60 haplotypes for the entire distribution range. The haplotype network did not show an apparent phylogeographical signal for the southern FMAs. Two populations were identified: Monte Hermoso (MH) and Necochea (NC)+Claromecó (CL)+Río Negro (RN). The low levels of genetic variability, the relative constant size over time, and the low levels of gene flow may indicate that MH has been colonized by a few maternal lineages and became isolated from geographically close populations. The apparent increase in NC+CL+RN size would be consistent with the higher genetic variability found, since genetic diversity is generally higher in older and expanding populations. Additionally, RN may have experienced a recent split from CL and NC; current high levels of gene flow may be occurring between the latter ones. FMA IV would comprise four franciscana dolphin populations: Samborombón West+Samborombón South, Cabo San Antonio+Buenos Aires East, NC+CL+Buenos Aires Southwest+RN and MH. Results achieved in this study need to be taken into account in order to ensure the long-term survival of the species.

## Introduction

Knowledge of population structure and patterns of gene flow are key components of management efforts, as they contribute to a better understanding of the ecology and the adaptive potential of species [1–4]. The degree of gene flow among coexisting populations may depend on different environmental and behavioral factors, such as the spatial separation between populations [5], the presence of physical barriers [6], the selection of a given habitat or reproductive pair [7], the specialization in a given resource [8] or a sex-biased dispersion [9], among others. As a result of stochastic and/or anthropogenic factors, the species population sizes may decline. If these populations become isolated, random genetic drift and inbreeding processes may be crucial in making them more vulnerable than larger ones to local extinction and the loss of genetic variation, resulting in a decrease in fitness [10,11].

The franciscana dolphin, *Pontoporia blainvillei*, is endemic to the Atlantic coast of South America. Its distribution extends from Itanúas (18°25’S) in Espíritu Santo (ES), Brazil, to Golfo San Matías (41°10’S) in northern Patagonia, Argentina [12], and is restricted within the 30 m isobaths from the coast [13–15]. The species has a small home range and a limited movement pattern, with a stepwise fashion moving between neighboring areas [15,16]. Like other coastal cetaceans, its distribution makes franciscana particularly vulnerable to anthropogenic activities, mainly to incidental by-catch [17–19]. Owing to its high incidental mortality in fishing gillnets [14,20–23], it is the most threatened small cetacean in the Southwestern Atlantic Ocean [24,25] and was classified as “Vulnerable” by the International Union for Conservation of Nature (IUCN) [26]. According to the International Whaling Commission Scientific Committee [27] a 1% incidental population mortality per year is a matter of concern in small cetacean populations, and a 2% mortality may not be sustainable. Annual mortality in franciscana dolphins reaches up to 2–5%, approximately (*e*.*g*. [14,15,22,28]), severely impacting on size and connectivity among populations, and may result in the loss of the species evolutionary potential [3,29].

**Fig 1:**
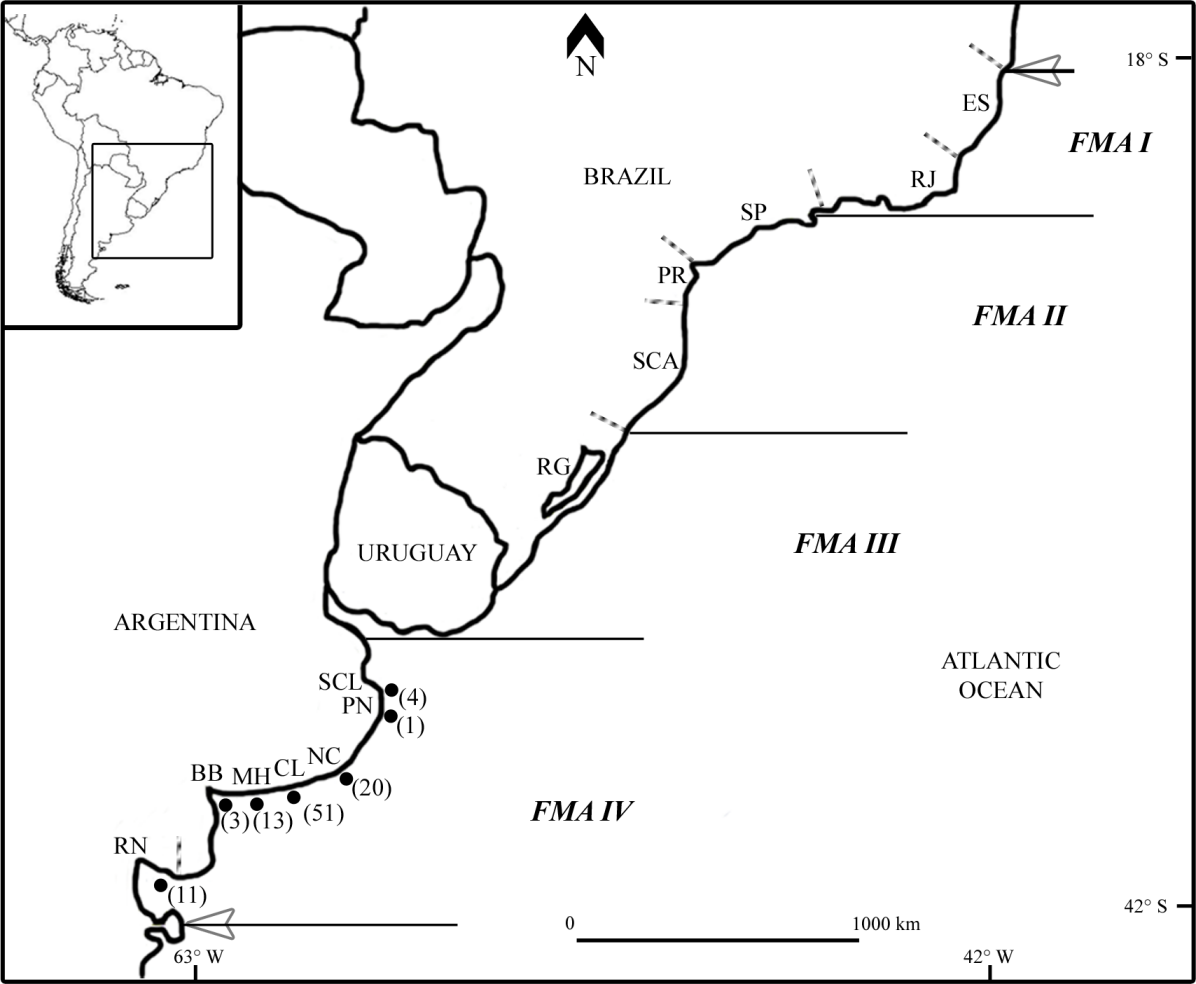
Franciscana Management Areas (FMAs) and sampled sites. Previously proposed FMAs (FMA I-VI) [24] are delineated with solid lines. The number of samples is shown between brackets. ES: Espíritu Santo; RJ: Rio de Janeiro; SP: São Paulo; PR: Paraná; SCA: Santa Catarina; RG: Rio Grande do Sul; SCL: San Clemente del Tuyú; PN: Pinamar; NC: Necochea; CL: Claromecó; MH: Monte Hermoso; BB: Bahía Blanca; RN: Río Negro. Note: Sample size for CL (N = 51) corresponds to 31 samples from Lázaro *et al*. [5] and 20 samples from this study.

Based on the species geographic distribution, contaminant and parasite loads, vital rates, and phenotype and genotype information available at that time, Secchi *et al*. [24] divided the species distribution range into four different segments called Franciscana Management Areas (FMAs): FMA I ranges from ES to Rio de Janeiro (RJ), FMA II from São Paulo (SP) to Santa Catarina (SCA), FMA III from Rio Grande do Sul (RG) to Uruguay (UY), whereas FMA IV includes the coasts of Buenos Aires and Río Negro (RN) in Argentina (Fig 1). Subsequently, studies based on mitochondrial DNA (mtDNA) analyses [3,5,30–32] confirmed Secchi’s subdivision and recognized the existence of San Clemente del Tuyú (SCL) [3], Argentina, as a genetically differentiated population. Additionally, Mendez *et al*. [33], performed genetic analyses including mtDNA and microsatellite information, suggesting the existence of 3 population within Argentina: Samborombón West (SW)/Samborombón South (SS), which includes SCL location; Cabo San Antonio (CSA)/Buenos Aires East (BAE); and Buenos Aires South (BAS)/Buenos Aires Southwest (BASW), which includes Claromecó (CL) location. However, although in Mendez *et al*. [3] no genetic differences were found between CSA and UY, Mendez *et al*. [33] did not compare CSA/BAE and UY. Based on this information, at least five genetic populations of franciscana dolphins have been distinguished: RJ, SP-Parana (PR); RG-UY, SW/SS and BAS/BASW; and a probable sixth population would be found in CSA/BAE. More recently, Cuhna *et al*. [34] performed a new genetic study in which they included sequences of franciscana dolphins from the northern limit of its range and re-assessed Secchi *et al*. [24] FMAs. They found additional genetically distinct populations in Brazil and an evolutionary break between franciscanas from the northern (ES to North RJ) and the southern (South RJ to Argentina) portions of its distribution, which indicate that they should be managed as independent Evolutionarily Significant Units. Additionally, Valsecchi & Zanelatto [32] found that the population from RJ, at the northern limit of the species distribution, showed the lowest genetic variability and suggested the analysis of this phenomenon in the southern populations of franciscana. Following Valsecchi & Zanelatto [32] suggestion, Negri [31] performed genetic analyses on 44 franciscana dolphins collected along the coastal area between Necochea (NC) and Bahía Blanca (BB), Argentina. However, the authors did not include in its analysis the population of franciscanas located off the coasts of RN, which includes the southernmost breeding area reported to date [35], providing only partial information about the southern populations of the species.

In this context, we further analyze the population genetic structure of the franciscana dolphin in the FMA IV, including samples from the southernmost portion of its geographic range.

## Materials and Methods

### Sample collection and DNA extraction

Tissue samples from 72 franciscana dolphins were collected from incidentally entangled and stranded franciscanas from seven localities along the coastal area between SCL and RN, Argentina (Fig 1). Sampling permits were issued by the Dirección de Fauna de la Provincia de Río Negro and the Dirección Provincial de Fiscalización y Uso Agropecuario de los Recursos Naturales (Ministerio de Asuntos Agrarios de la Provincia de Buenos Aires), Argentina. All tissue samples were preserved in 96% ethanol and/or 20% dimethyl sulfoxide (DMSO). Total DNA was extracted from samples using a proteinase K digestion, extraction of proteins with a phenol-chloroform method and alcohol precipitation of DNA [36].

### Mitochondrial DNA control region sequencing

A fragment of approximately 530 bp from the mtDNA control region was amplified by polymerase chain reaction (PCR) using primers THR L15926 5´ TCA AAG CTT ACA CCA GTC TTG TAA ACC [37] and TDKD 5´ CCT GAA GTA GGA ACC AGA TG [38].

Final concentrations used in PCR reaction volumes of 50 μl were: 5 μg/ml template DNA, Buffer 1X (Promega), 0.2 mM dNTPs, 0.2 μM each primer, 1.5 mM MgCl_2_ and 1.25 units of GoTaq polymerase (Promega). PCR cycling profile consisted of an initial denaturation at 94°C for 2 min, followed by thirty-seven cycles of denaturation at 94°C for 1 min, anneling at 47°C for 1 min and polymerase extension at 72°C for 1 min, and a final extension at 72°C for 5 min. PCR products were purified using a commercial kit (AccuPrep PCR Purification Kit, Bioneer) and sequenced in both directions using an ABI 337 Automated DNA Prism Sequencer (Applied Biosystems, Inc.). When new mtDNA haplotypes were found, we performed this procedure at least twice to confirm the results.

### Data analysis

CLUSTALX 2.0.11 [39] was used to align DNA sequences and to identify polymorphic sites. The mtDNA haplotypes were compared with those previously published for the species (SA-SK [40], L1-L22 [5], M1-M19 [3], N1 and N3 [31], C23-C28 [30], CU1-2 and CU4-CU7 [34]). Haplotypes were verified using DnaSP v5.10.01 [41]. In order to study patterns of geographical distribution and haplotype relationships, a Median-Joining network [42] was implemented in Network 4.6.1.1 (Fluxus Technology Inc.). To remove all superfluous median vectors and links that were not contained in the shortest tree of the network, reducing network complexity, a Maximum-Parsimony post-processing was conducted [43].

In order to further evaluate the southernmost portion of the species range, we analyzed samples from the localities of NC, CL, MH and RN (Fig 1). Samples collected from SCL (n = 4), Pinamar (PN) (n = 1) and Bahía Blanca (BB) (n = 3) were not included in the analysis due to the small sample size. Also, genetic data from some studies within FMA IV [3,33] could not be included as the haplotype frequencies or their exact sample collection site were not reported.

Haplotype (h) and nucleotide diversity (π) of the data set were assessed using Arlequin v3.5 [44].

For the analysis of population structure we performed an Analysis of Molecular Variance (AMOVA) using Arlequin v3.5 [44]. Since in a previous study [33], genetic differences were found between geographically close populations, and considering that the artisanal fisheries in our sampled locations tend not to overlap, we defined 4 populations: NC, CL, MH and RN. Population pairwise F_ST_ values were analyzed using Arlequin v3.5 [44]. We also performed a Mantel test without grouping populations in order to test for isolation by distance (IBD). The correlation was examined between F_ST_/(1-F_ST_) and the logarithm the geographical distance between sites using IBD v3.23 [45]. Geographical distances between locations, measured as the minimum distance by sea between each other, were calculated using a Geographic Information System (GIS) in ArcGIS software. As evidence of IBD, the rejection of the null hypothesis of a flat or negative slope between genetic and geographical distances was used.

In order to study the historical demography of the species, we analyzed the distribution of the observed number of pairwise differences among all haplotypes in a sample, or a mismatch distribution analysis [46,47] for each of the populations obtained from the population pairwise genetic analysis (see below). Goodness of fit between the observed and expected mismatch was assessed by the Harpending’s raggedness index (*r*) [48]. This index quantifies the smoothness of the observed pairwise difference distribution and a nonsignificant result indicates a good fit to a population expansion model [48]. Typically, populations that had undergone a recent expansion show smooth and unimodal distributions; bimodal distribution patterns are suggestive of two expansions at different times; and populations that had been stationary for a long time show ragged and multimodal distributions [48–51]. Additionally, Tajima’s *D* [52] and Fu’s *F*_*S*_ [53] neutrality tests were performed. Both tests were developed to detect departures of DNA polymorphisms from the neutral expectations. Tajima’s *D* [52] uses the frequency of segregating nucleotide sites and Fu’s *F*_*S*_ [53] uses the haplotypes distribution. Significantly negative values of Tajima’s *D* [52], due to an excess of rare alleles, indicate population expansion or selective sweep, whereas significantly positive values, due to an excess of intermediate frequency alleles, indicate genetic subdivision or diversifying selection. Large negative values of Fu’s *F*_*S*_ [53], due to an excess of rare alleles, indicate population growth or genetic hitchhiking. All of these analyses were accomplished using DnaSP v5.10.01 [41] and Arlequin v3.5 [44]. Additionally, migration rates and divergence times between putative populations (NC-CL, NC-MH, NC-RN, CL-MH, CL-RN and MH-RN) were obtained with a Markov Chain Monte Carlo (MCMC) approach as implemented in the program MDIV [54]. The program estimates the parameter theta, which is a product of the effective population size and the mutation rate of the studied gene region (*θ = 4N*_*e*_μ), the migration rate per gene per generation between populations scaled by the effective population size (*M = 2N*_*e*_ *m*), and the time since the two populations diverged scaled by the effective population size (*T = t/2N*_*e*_). We used the finite sites (HKY) model and performed 10 independent runs of 2 x 10^6^ iterations each and a burn-in of 5 x 10^5^ iterations. Likelihood values for each parameter were estimated and those with the highest posterior probability were accepted as the best estimates. We further analyzed patterns of historical demography with the Bayesian skyline plot method of Drummond *et al*. using BEAST 1.6 [55]. This model, that uses standard MCMC sampling procedures, provides a powerful framework for estimating effective population size through time. The method produces credibility intervals that represent the combined phylogenetic and coalescent uncertainty [56]. Coalescent reconstructions used a strict molecular clock with a substitution rate of 1.83 x 10^−2^ substitutions/site/My [57], the HKY+G+I model of mutation, as indicated by JModelTest [58], and five grouped intervals. Three replicates of 4 x 10^7^ MCMC steps each were run. The first 10% of each run was discarded as burn-in. Results were checked for convergence to a stationary distribution in Tracer 1.6 and combined using LogCombiner 1.6. Higher estimates of molecular evolutionary rates based on population studies than those inferred from phylogenetic studies have been previously described (*e*.*g*. [59,60]). Since we used a rate estimated from a phylogenetic study for the families of the river dolphins Iniidae and Pontoporiidae [57], time estimates for our populations may be overestimated due to the time dependency of molecular evolutionary rates.

## Results

### Franciscana dolphin haplotypes

From the analysis of the mtDNA control region of the 72 samples collected in this study, we detected 22 haplotypes, including 5 novel ones (GenBank accession numbers: KP670446 to KP670450), totalizing 60 haplotypes for the entire distribution range of the species (S1 Table). We also found that haplotype CU1 reported in Cunha *et al*. [34] was a shorter version of haplotype SD [40] and, as previously reported [30], haplotypes M4, M5 and M12 [3] were shorter sequences of another previously reported haplotype (J [40] and L3 [5]). Also, haplotype CU5 [34] was a longer version of haplotype C28 [30]. Although several samples analyzed here were those used in Negri [31], we did not find haplotype N1 in our data set, and haplotype N3 was found in CL, not in NC as it was reported in that study (S1 Table).

### Phylogeographic analysis and genetic variation

**Fig 2:**
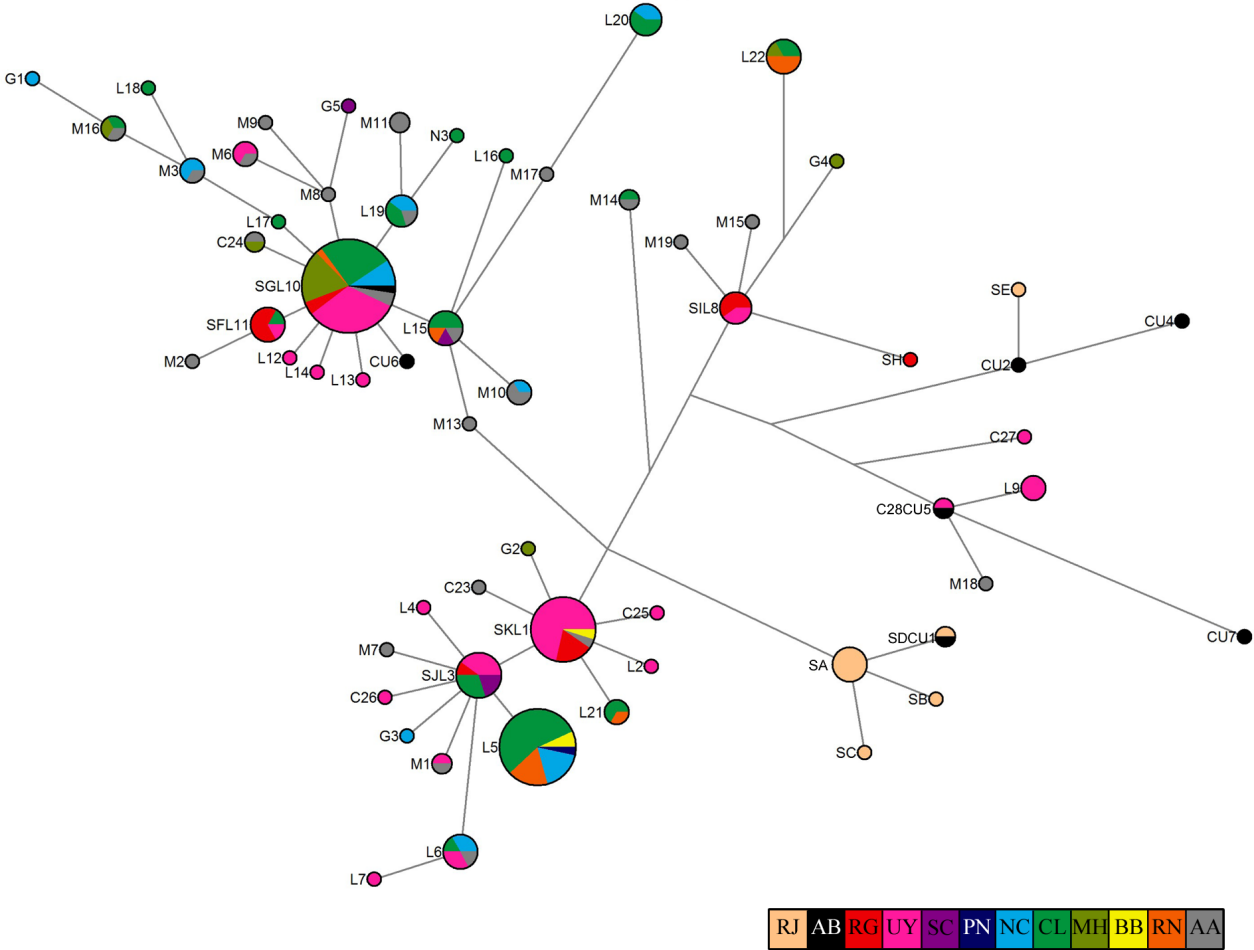
Median-joining network based on the mtDNA control region haplotypes of franciscana dolphins. The size of the circle is proportional to frequency. Branch length reflects the number of mutations separating any two haplotypes. AA and AB: haplotypes with unknown exact sampling site and/or frequency, collected from Argentina [3,33] and Brazil [34], respectively. The frequency for AA and AB was set to one individual. RJ: Rio de Janeiro; RG: Rio Grande do Sul; UY: Uruguay; SCL: San Clemente del Tuyú; PN: Pinamar; NC: Necochea; CL: Claromecó; MH: Monte Hermoso; BB: Bahía Blanca; RN: Río Negro. Haplotype SJ-L3-M4-M5-M12 is shown with a shorter nomenclature, SJL3.

The complex phylogeographic relationship among haplotypes is shown in Fig 2. It uncovered 3 main groups of haplotypes, composed of 4 to 26 haplotypes. The most geographically expanded group is composed of 26 haplotypes found from SP [34] to RN. Haplotype SG-L10 is the most common of the group, and many other haplotypes connect to it in a star-like topology. The second group comprised 15 haplotypes found from RG [5] to RN. In this group, haplotype SJ-L3 and, to a lesser extent, haplotype SK-L1, showed a star shaped topology. The only phylogeographic signal was observed in the third group, composed of 4 haplotypes (SA-SD) and found only in the northernmost localities of the species distribution range (ES and North RJ) [34,40]. The other haplotypes, including one of the novel ones, are scattered throughout the species distribution range.

From the analysis of intrapopulations genetic variability, averaged haplotype and nucleotide diversity indexes observed were 0.786 (±0.111) and 0.012 (±0.003), respectively. MH showed the lowest haplotype and nucleotide diversity values, and NC the highest (Table 1).

**Table 1:** Genetic diversity indexes and neutrality tests estimates for each locality and population, respectively. N: sample size; n: number of haplotypes; h: haplotype diversity; π: nucleotide diversity. NC: Necochea; CL: Claromecó; MH: Monte Hermoso; RN: Río Negro.

*Locality*	*N*	*n*	*h*	*π*	*Tajima*’*s D* *	*Fu*’*s F*_*S*_ *
NC	20	9	0.895	0.014		
CL	51	16	0.845	0.013	0.138	-1.839
RN	11	5	0.764	0.014		
MH	13	6	0.641	0.008	-0.411	-0.129

*Neutrality tests statistics were not statistically significant (*P* > 0.3).

### Population differentiation

The AMOVA showed a global significant difference between localities (F_ST_ = 0,054; *P* = 0.001); the greatest source of variation (94,6%) was found within localities. In the pairwise comparisons, significant differences were found between MH and all other localities, but not between the latter ones (Table 2).

**Table 2:** Pairwise genetic differentiation between putative populations.

	*F*_*ST*_ *	*P*
NC/CL	-0.007	0.592
NC/MH	**0.117**	0.002
NC/RN	0.041	0.117
CL/MH	**0.112**	10^−4^
CL/RN	0.011	0.260
MH/RN	**0.241**	0.001

* Significant values at *P* < 0.01 are shown in bold.

A negative and non-significant correlation between genetic and geographical distances was observed when the Mantel Test was performed (r = -0.040, *P* = 0.588) (S1 Fig).

### Demographic trends

Migration rates estimates were consistent with genetic distances (S2 Table). The highest migration rate was obtained between CL-NC and the lowest between MH-RN. In general, migration rates and divergence times were inversely related, as expected (S2 Table).

**Fig 3:**
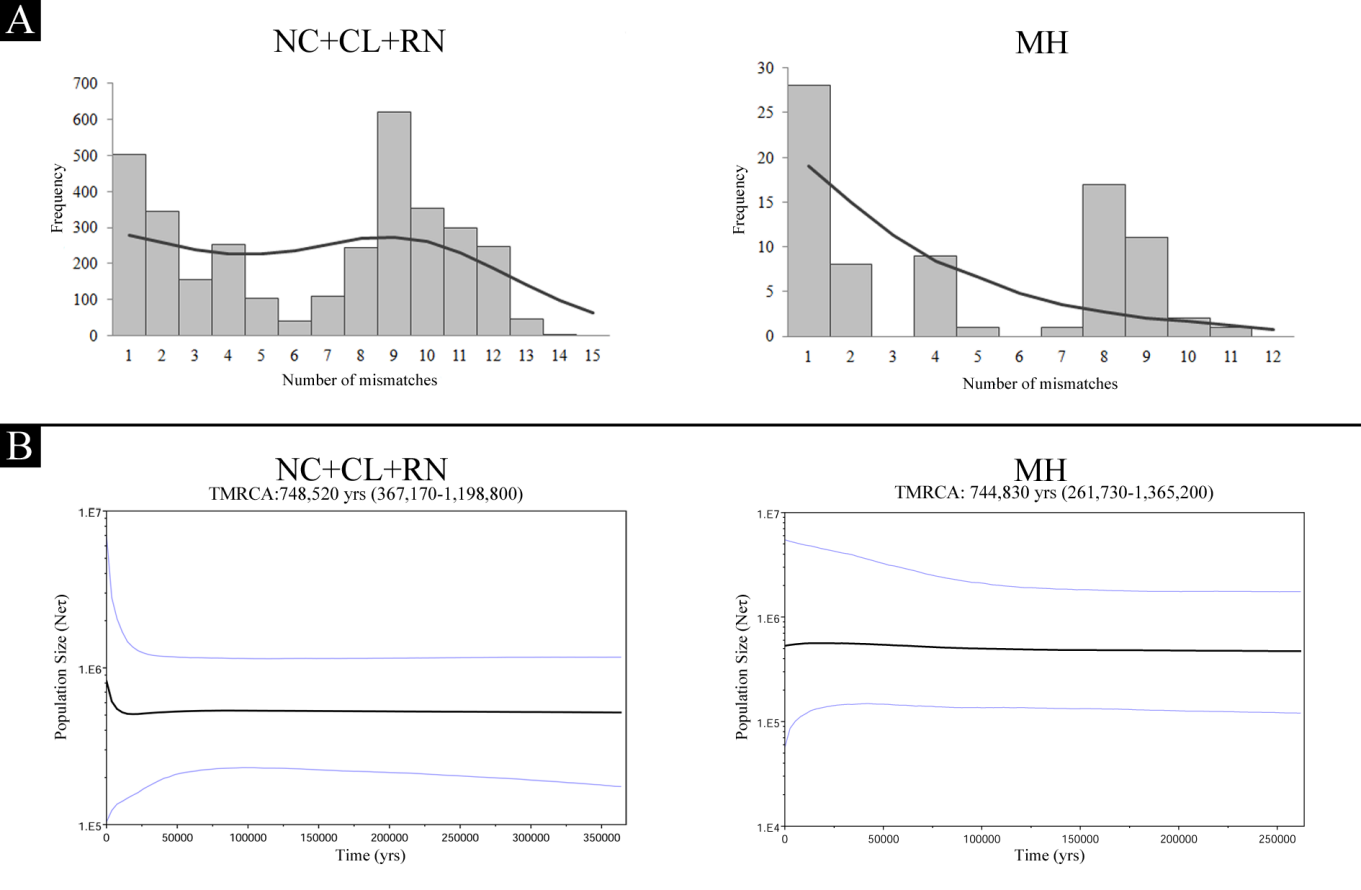
Demographic history based on the mtDNA control region sequences from populations NC+CL+NC and MH. A) Mismatch distributions. Observed and expected distributions are shown with bars and lines, respectively. B) Bayesian skyline plots. The black line is the median estimated and the blue lines show the 95% highest posterior density (HPD) intervals. NC: Necochea; CL: Claromecó; RN: Río Negro; MH: Monte Hermoso.

When demographic history was analyzed based on the mismatch distribution, populations differed among their demographic histories. Using goodness of fit tests based on the *r*, the adequacy of the sudden expansion model could not be rejected for NC+CL+RN (*P* = 0.15 for NC+CL+RN; *P* = 0.02 for MH). Furthermore, the distribution obtained for this population showed a bimodal graph, suggestive of two expansions at different times, with a dominant right wave crest translated to an estimated time since expansion of approximately 631,500 years before present (ybp) (Fig 3). In contrast, as expected under a model of relative constant population size, MH showed a multimodal mismatch distribution (Fig 3). Also, for both populations non-departure from the null hypothesis of neutrality was observed (Table 1).

Furthermore, based on the Bayesian skyline plot results MH and NC+CL+RN seem to have kept a stable size (Fig 3).

## Discussion

Genetic studies are important in order to develop effective management and conservation strategies to ensure the long-term survival of the species. In this study, we have collected and analyzed genetic information of franciscana dolphins from the southern management area (FMA IV), including samples from the southernmost edge of the species geographical range (RN), describing new haplotypes and contributing to enlarging our knowledge of the species population genetics.

### Haplotype identity

Previous studies of the genetic structure in franciscana populations based on mtDNA analysis reported 25 haplotypes as new [3,34]. From these, 5 proved to be shorter or longer versions of already reported types (see Results). Based on the revision of published haplotypes and the results obtained in this work, in which 5 novel haplotypes were found, a total of 60 haplotypes are currently reported for the entire distribution range of the franciscana dolphin (S1 Table).

### Population structure

Our results showed evidence for the existence of at least two genetically distinct populations within FMA IV: NC+CL+RN and MH (Table 2). Since Mendez *et al*. [33] BAS location was composed of 8 samples from NC and the 31 samples from CL [5] that we also included in our 51 analyzed samples from CL, and as we collected samples from NC (N = 20) as well, our NC+CL+RN population would correspond to the same one, originally recognized by Lázaro *et al*. [5] and later by Mendez *et al*. [33]. Furthermore, because many of the haplotypes reported for the northern locations of Buenos Aires [3,33] were not found in our study, four populations of franciscana dolphin would be comprised within FMA IV: SW+SS, CSA+BAE, NC+CL+BASW+RN and MH.

The genetic population structure of many species is characterized by a pattern of IBD, since the potential dispersal of individuals tends to diminish with the geographical distances [61,62]. In previous studies based on mtDNA analyses, genetic differentiation between the franciscana dolphin populations could be explained by geographical distances [5,33]. However, in the southernmost area of the species distribution, our Mantel Test did not support the existence of IBD (S1 Fig). Although IBD can lead to population differentiation over a species distribution, across small geographical areas, other factors may be important drivers of genetic differentiation [63]. In this study, we found existence of genetic differences between MH and neighboring sampling sites (NC and CL), but not between more distant ones (NC-RN and CL-RN) (Table 2). Resource specialization may lead to intraspecific genetic differentiation among some cetacean populations [64]. Many cases of genetic divergences due to resource specialization among cetacean populations had been documented, such as those found in resident and transient killer whales (*Orcinus orca*) in the Eastern North Pacific [65], long-finned pilot whales (*Globicephala melas*) in the North Atlantic [66], inshore and offshore populations of bottlenose dolphins (*Tursiops truncates*) in the Gulf of Mexico [7], among other examples. Along its geographic distribution, the franciscana dolphin populations are found in a variable range of marine habitats, from estuarine to open ocean ones, which could lead to local specializations. Based on genetic and environmental data, Mendez *et al*. [33] proposed that isolation by spatial distance is not the only mechanism acting in the species population structuring, but also processes of isolation by environmental distances may play an important role among contiguous populations within the northern area of FMA IV. In the southern populations of FMA IV, Paso Viola [67] found differences in the diet between NC, CL and MH; while NC and CL populations feed primary on *Loligo sanpaulensis*, MH majority preys are *Cynoscion guatucupa* and *Artemesia longinaris*. This could indicate a possible resource specialization in the southern populations which, in turn, may contribute to the observed MH differentiation. However, a more thorough study including environmental and diet analyses is needed in the southern portion of the franciscana distribution range in order to test this hypothesis.

### Phylogeography and demographic tendency

Despite the addition of new samples collected in this study, and in agreement with previous findings [3,5,30], haplotype SG-L10 was the most frequent and widespread along franciscana distribution, and several others haplotypes connect to it showing a star-shaped topology (Fig 2). This phylogeographic pattern suggests that haplotype SG-L10 would be an ancestral haplotype from which other haplotypes derived due to a rapid radiation from an ancient population [68–70].

High levels of haplotype and nucleotide diversity were found in NC and CL, with NC presenting the highest ones (Table 1). Similar values were reported for other marine dolphins, such as the coastal *Sotalia* dolphins [71] and *Phocoena phocoena* [72]. When analyzing our results with those previously published along the franciscana distribution [3,5,30,32–34,40], overall higher levels of genetic variability were found in south-central populations. Since genetic diversity is generally higher in older and expanding populations [73,74], the observed diversity pattern would support the hypothesis of a colonization of the Southwestern Atlantic from the south northwards, as previously proposed [34,75].

In MH, low levels of genetic diversity were found (Table 1), comparable to those observed in BASW [33]. Additionally, our results also suggest historical low levels of gene flow between MH and the other analyzed localities (S2 Table) and a relative constant size over time (Fig 3). A possible interpretation for these results is that MH may have been colonized by few maternal lineages [74], and became isolated from geographically close populations due to specialization over a limited resource (see above).

Between NC, CL and RN, high levels of gene flow and a lack of genetic differentiation were observed (S2 Table and Table 2, respectively). Similar results were obtained for BAS and BASW [33]. Two plausible explanations could be proposed for these results: current high levels of gene flow between localities or a recent split between them [69,76]. Due to its relative close geographical distance and considering a similar resource specialization [67], the former interpretation is more likely to explain NC-CL results. In contrast, due to the species small home range and limited movement patterns [16,77], the reported year-round presence of the species in RN [78] and the identification of RN as the southernmost breeding site for the species [35], the latter explanation is more plausible to explain the results regarding RN.

However, demographic tendencies should be regarded as preliminary since they were presented analyzing one locus. Analyses involving additional loci and samples would be necessary to support our results.

### Conclusions

Our analysis shows the existence of two genetically different populations within FMA IV: NC+CL+RN and MH. Considering Mendez *et al*. [3,33] results, FMA IV would comprise four populations: SW+SS, CSA+BAE, MH and NC+CL+BASW+RN. The existence of a division within FMA IV may be due to a combination of resource specialization and isolation by distance [3,33,67]. While SW/SS and MH would be estuarine populations, CSA+BAE and NC+CL+BASW+RN would be oceanic ones.

Conservation plans and management efforts should take this separation into account in order to ensure the long-term survival of the species. Particularly the low levels of genetic diversity found in MH, should be a source of concern as genetic variation is usually assumed to be critical for the long-term viability of the species [79,80]. Additionally, the status of RN should be further analyzed, since this location encompasses the southernmost breeding site of the franciscana dolphin [35] and presents a year-round presence of the species [78]. Further analyses involving other loci and environmental information would be necessary in order to enhance our results.

## Supporting Information

S1 Fig Genetic isolation by distance. Results of the Mantel test for correlation between the genetic distance [F_ST_/(1-F_ST_)] and the logarithm (Log) of geographic distance between sampling sites.
(TIF)

S1 Table Frequency of occurrence and sampling sites of the mtDNA control region haplotypes. Asterisks show the novel haplotypes found in this study. Dashes indicate unknown frequency. AA and AB: haplotypes with unknown exact sampling site and/or frequency, collected from Argentina [3,30] and Brazil [34], respectively. RJ: Rio de Janeiro; RG: Rio Grande do Sul; UY: Uruguay; SCL: San Clemente del Tuyú; PN: Pinamar; NC: Necochea; CL: Claromecó; MH: Monte Hermoso; BB: Bahía Blanca; RN: Río Negro.
(DOCX)

S2 Table Gene flow between localities. Estimates of migration rates, time since divergence and θ between locations. NC: Necochea; CL: Claromecó; MH: Monte Hermoso; RN: Río Negro.
(DOCX)
