# Supplementary figures and images for: Population Genetics of Franciscana Dolphins (Pontoporia blainvillei): Introducing a New Population from the Southern Edge of Their Distribution
Source: PLoS One. 2015 Jul 29;10(7):e0132854. doi: 10.1371/journal.pone.0132854 (PMC4519281; doi:10.1371/journal.pone.0132854)

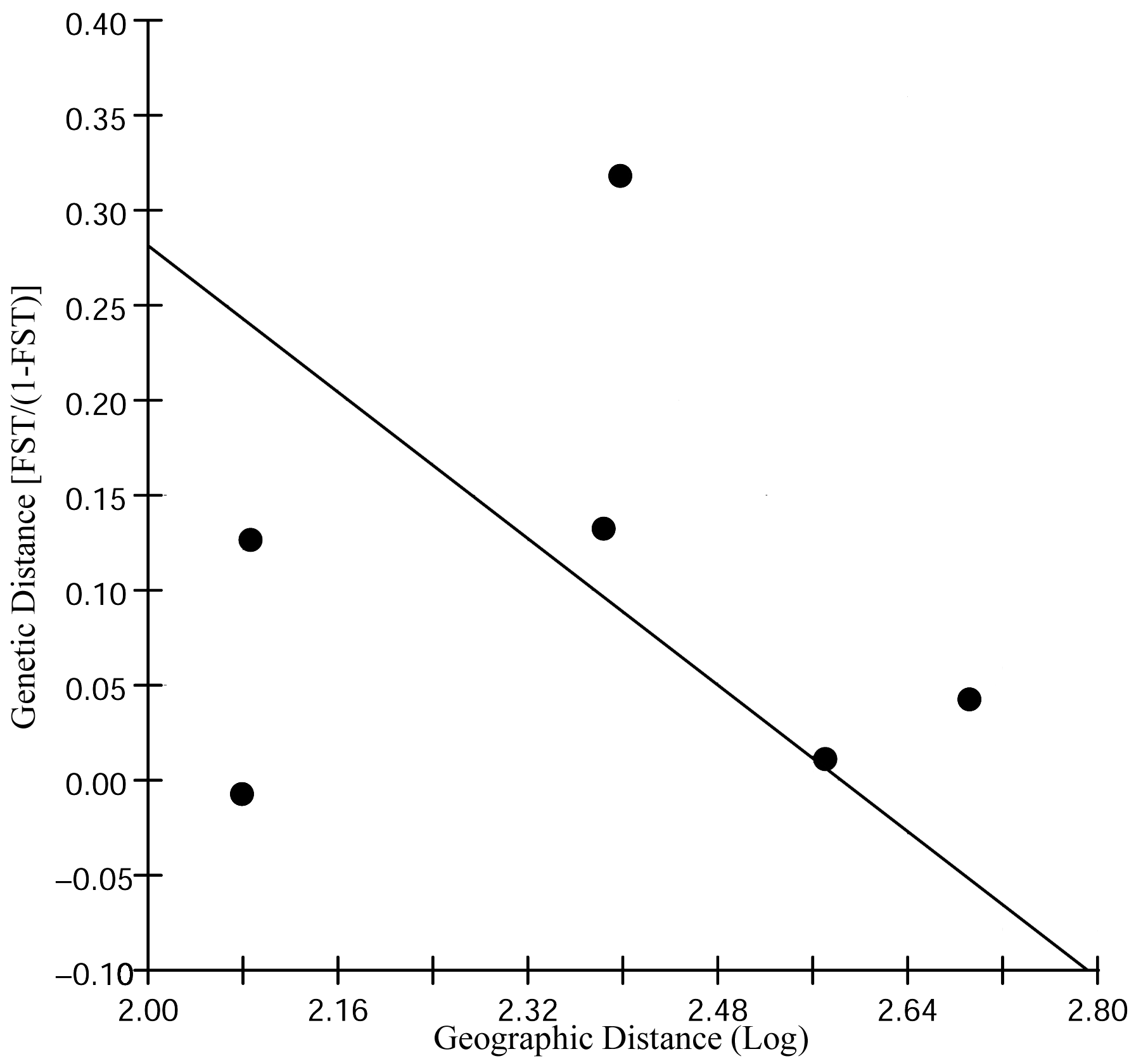

Supplement: S1 Fig — Results of the Mantel test for correlation between the genetic distance [FST/(1-FST)] and the logarithm (Log) of geographic distance between sampling sites. (TIF) [file pone.0132854.s001.tif]
