# Supplementary material for: Population Genetics of Franciscana Dolphins (Pontoporia blainvillei): Introducing a New Population from the Southern Edge of Their Distribution
Source: PLoS One. 2015 Jul 29;10(7):e0132854. doi: 10.1371/journal.pone.0132854 (PMC4519281; doi:10.1371/journal.pone.0132854)
Supplement: S1 Table — Asterisks show the novel haplotypes found in this study. Dashes indicate unknown frequency. AA and AB: haplotypes with unknown exact sampling site and/or frequency, collected from Argentina [3,30] and Brazil [34], respectively. RJ: Rio de Janeiro; RG: Rio Grande do Sul; UY: Uruguay; SCL: San Clemente del Tuyú; PN: Pinamar; NC: Necochea; CL: Claromecó; MH: Monte Hermoso; BB: Bahía Blanca; RN: Río Negro. (DOCX) [file pone.0132854.s002.docx]

| **HAPLOTYPE** | **RJ** | **AB** | **RG** | **UY** | **AA** | **SCL** | **PN** | **NC** | **CL** | **MH** | **BB** | **RN** |
| --- | --- | --- | --- | --- | --- | --- | --- | --- | --- | --- | --- | --- |
| SA-CU1 | 6 | - |  |  |  |  |  |  |  |  |  |  |
| SB | 1 |  |  |  |  |  |  |  |  |  |  |  |
| SC | 1 |  |  |  |  |  |  |  |  |  |  |  |
| SD | 1 |  |  |  |  |  |  |  |  |  |  |  |
| SE | 1 |  |  |  |  |  |  |  |  |  |  |  |
| SH |  |  | 1 |  |  |  |  |  |  |  |  |  |
| SK-L1 |  |  | 4 | 15 |  |  |  |  |  |  | 1 |  |
| L2 |  |  |  | 1 |  |  |  |  |  |  |  |  |
| SJ-L3-M4-M5-M12 |  |  | 1 | 4 | - | 2 |  |  | 3 |  |  |  |
| L4 |  |  |  | 1 |  |  |  |  |  |  |  |  |
| L5 |  |  |  |  |  |  | 1 | 5 | 16 |  | 2 | 5 |
| L6 |  |  |  | 2 |  |  |  | 2 | 1 |  |  |  |
| L7 |  |  |  | 1 |  |  |  |  |  |  |  |  |
| SI-L8 |  |  | 3 | 2 |  |  |  |  |  |  |  |  |
| L9 |  |  |  | 3 |  |  |  |  |  |  |  |  |
| SG-L10 |  |  | 2 | 14 |  |  |  | 4 | 12 | 8 |  | 1 |
| SF-L11 |  |  | 4 | 1 |  |  |  |  | 1 |  |  |  |
| L12 |  |  |  | 1 |  |  |  |  |  |  |  |  |
| L13 |  |  |  | 1 |  |  |  |  |  |  |  |  |
| L14 |  |  |  | 1 |  |  |  |  |  |  |  |  |
| L15 |  |  |  |  |  | 1 |  |  | 3 |  |  | 1 |
| L16 |  |  |  |  |  |  |  |  | 1 |  |  |  |
| L17 |  |  |  | 1 |  |  |  |  | 1 |  |  |  |
| L18 |  |  |  |  |  |  |  |  | 1 |  |  |  |
| L19 |  |  |  |  |  |  |  | 2 | 2 |  |  |  |
| L20 |  |  |  |  |  |  |  | 2 | 3 |  |  |  |
| L21 |  |  |  |  |  |  |  |  | 2 |  |  | 1 |
| L22 |  |  |  |  |  |  |  |  | 2 | 1 |  | 3 |
| M1 |  |  |  | 1 | - |  |  |  |  |  |  |  |
| M2 |  |  |  |  | - |  |  |  |  |  |  |  |
| M3 |  |  |  |  | - |  |  | 2 |  |  |  |  |
| M6 |  |  |  | 2 | - |  |  |  |  |  |  |  |
| M7 |  |  |  |  | - |  |  |  |  |  |  |  |
| M8 |  |  |  |  | - |  |  |  |  |  |  |  |
| M9 |  |  |  |  | - |  |  |  |  |  |  |  |
| M10 |  |  |  |  | - |  |  | 1 |  |  |  |  |
| M11 |  |  |  |  | - |  |  |  |  |  |  |  |
| M13 |  |  |  |  | - |  |  |  |  |  |  |  |
| M14 |  |  |  |  | - |  |  |  | 1 |  |  |  |
| M15 |  |  |  |  | - |  |  |  |  |  |  |  |
| M16 |  |  |  |  | - |  |  |  | 1 | 1 |  |  |
| M17 |  |  |  |  | - |  |  |  |  |  |  |  |
| M18 |  |  |  |  | - |  |  |  |  |  |  |  |
| M19 |  |  |  |  | - |  |  |  |  |  |  |  |
| C23 |  |  |  |  | - |  |  |  |  |  |  |  |
| C24 |  |  |  |  | - |  |  |  |  | 1 |  |  |
| C25 |  |  |  | 1 |  |  |  |  |  |  |  |  |
| C26 |  |  |  | 1 |  |  |  |  |  |  |  |  |
| C27 |  |  |  | 1 |  |  |  |  |  |  |  |  |
| C28-CU5 |  | - |  | 1 |  |  |  |  |  |  |  |  |
| CU2 |  | - |  |  |  |  |  |  |  |  |  |  |
| CU4 |  | - |  |  |  |  |  |  |  |  |  |  |
| CU6 |  | - |  |  |  |  |  |  |  |  |  |  |
| CU7 |  | - |  |  |  |  |  |  |  |  |  |  |
| N3 |  |  |  |  |  |  |  |  | 1 |  |  |  |
| G1* |  |  |  |  |  |  |  | 1 |  |  |  |  |
| G2* |  |  |  |  |  |  |  |  |  | 1 |  |  |
| G3* |  |  |  |  |  |  |  | 1 |  |  |  |  |
| G4* |  |  |  |  |  |  |  |  |  | 1 |  |  |
| G5* |  |  |  |  |  | 1 |  |  |  |  |  |  |
