# Supplementary material for: Population Genetics of Franciscana Dolphins (Pontoporia blainvillei): Introducing a New Population from the Southern Edge of Their Distribution
Source: PLoS One. 2015 Jul 29;10(7):e0132854. doi: 10.1371/journal.pone.0132854 (PMC4519281; doi:10.1371/journal.pone.0132854)
Supplement: S2 Table — Estimates of migration rates, time since divergence and θ between locations. NC: Necochea; CL: Claromecó; MH: Monte Hermoso; RN: Río Negro. (DOCX) [file pone.0132854.s003.docx]

|  | **Θ = 4N*_e_µ*** | **M *= 2N_e_m*** | **T *= t/2N_e_*** |
| --- | --- | --- | --- |
| CL-NC | 4.39 | 32.47 | 0.06 |
| CL-MH | 4.81 | 2.46 | 0.29 |
| CL-RN | 3.95 | 19.94 | 0.06 |
| NC-MH | 4.77 | 1.93 | 0.33 |
| NC-RN | 3.82 | 6.47 | 0.07 |
| MH-RN | 3.02 | 1.08 | 0.28 |
